# Prevalence and risk factors of pre-sick building syndrome: characteristics of indoor environmental and individual factors

**DOI:** 10.1186/s12199-019-0830-8

**Published:** 2019-12-17

**Authors:** Yoshitake Nakayama, Hiroko Nakaoka, Norimichi Suzuki, Kayo Tsumura, Masamichi Hanazato, Emiko Todaka, Chisato Mori

**Affiliations:** 1 Center for Preventive Medical Sciences, Chiba University, 6-2-1 Kashiwanoha, Kashiwa, Chiba, 277-0882 Japan; 2 Department of Bioenvironmental Medicine, Graduate School of Medicine, Chiba University, Chiba, Japan

**Keywords:** Air quality, Sick building syndrome, Residential environment, Lifestyle, Online survey

## Abstract

**Background:**

With the aim to prevent sick building syndrome and worsening of allergic symptoms, primarily resulting from the indoor environment, the relationships among people’s residential environment in recent years, their lifestyle habits, their awareness, and their symptoms were investigated using an online survey.

**Methods:**

In the survey, respondents experiencing symptoms specific to sick building syndrome, although they were not diagnosed with sick building syndrome, were categorized in the pre-sick building syndrome group. The relationships among individual characteristics, residential environment, and individual awareness were analyzed.

**Results:**

Results showed that the prevalence of pre-sick building syndrome was high among young (aged 20–29 years) population of both sexes. In addition, “condensation,” “moisture,” “musty odors” in the house, and the “use of deodorant and fragrance” were all significantly associated with pre-sick building syndrome. Conversely, there was no significant association with recently built “wooden” houses that are highly airtight and have thermal insulation.

**Conclusions:**

Efficient “ventilation” plans and “ventilation” improvement and air conditioning systems to prevent mold and condensation in rooms are necessary to maintain a good, indoor environment that is beneficial for health. Efforts should also be made to encourage individuals to regularly clean and effectively ventilate their homes.

## Background

Till date, many studies have been globally conducted to investigate indoor chemical substances and their influence on health to prevent “sick building syndrome (SBS);” the symptoms of “SBS” include mucosal irritation and allergy-like symptoms and are linked to spending time in a specific building [1–11]. In Japan, restrictions have been placed on the concentration of certain volatile organic compounds (VOCs) and total volatile organic compounds generated by implementing values in the indoor concentration guideline by the Ministry of Health, Labour and Welfare, and by the revisions of the building-standard laws by the Ministry of Land, Infrastructure and Transport [12, 13]. However, “SBS” is still persistent. In 2017, the “Manual for Consultation and Countermeasures on Sick House Syndrome Based on Scientific Evidence (revised new edition)” was created by the Ministry of Health, Labour and Welfare Research Group [14], and in 2019, the Ministry of Health, Labour and Welfare further revised the values for xylene, di-n-butyl phthalate, and di(2-ethylhexyl)phthalate concentrations in the guidelines [15]. Various indoor factors cause physical ailments, including chemical factors, such as exposure to chemical substances volatilizing from building materials, furniture, and household goods; biological factors, such as mold, ticks, and bacteria; physical factors, such as heat, light, noise, and radiation; and social and psychological factors of residents [16–18]. “SBS” is associated with the awareness of residents to improve their indoor air quality by opening windows for “ventilation,” cleaning, and lifestyle and with the air environment such as indoor chemical substance concentrations, temperature, humidity, and odor [19, 20]. In addition, recently, owing to the development of new, highly insulated, airtight housing, there is a growing concern of an increase in living spaces where chemical substances are more likely to accumulate compared with conventional houses. Furthermore, a growing, global appreciation for “wooden” architecture and global interest in cross-laminated timber [21], in conjunction with the enforcement of a law by the Japanese government that promotes the use of wooden materials in public buildings [22], may increase the risk of generating unregulated chemical substances due to the development of new building materials, adhesives, paints, and construction techniques [23, 24]. Although studies regarding indoor environments and the onset of “SBS” or allergic symptoms have been conducted in the past [25–27], with changes in the housing environment over time, it is important to continue to investigate and assess the impacts of indoor environments on health. Therefore, we conducted a comprehensive survey using an online questionnaire regarding the occurrence and risk factors of “SBS” to investigate the association between indoor environment accompanying the aforementioned changes in residential environments over time, the characteristics of residents and their awareness regarding air quality and diseases, and “SBS” and allergy-like symptoms, at the Center for Preventive Medical Sciences, Chiba University, in June 2017. The aims of this study are first, to collect data on the relationship between recent living environment and lifestyle as well as “SBS” and allergy-like symptoms; second, to define pre-SBS according to respondents’ experience and the frequency of “SBS” symptoms; and third, to statistically analyze the effects of those environmental and personal factors on pre-SBS people.

## Methods

### Data collection

A nationwide, questionnaire survey was administered to 1500 individuals (750 males and 750 females; age 18–70 years). The survey was implemented over 4 days from June 16 to June 19, 2017. All participants were provided the information that by answering the survey, they will be deemed to consent to cooperate with this study. The survey was conducted by a web research service (Rakuten Research Co., Ltd.) that used random sampling after screening for sex, age, and location of residence. Data from 1500 participants with no missing items were collected and analyzed.

### Questionnaires

The questionnaire comprised 31 questions in total: 13 regarding participant characteristics; 4 regarding SBS-specific symptoms; 12 regarding residential environments, specifically the indoor environment of buildings where participants spent most of their day and had the largest impact on their health; and 2 regarding respondent’s awareness of air quality. Questions regarding participant characteristics related to age, sex, years of education, “family income,” medical history [“asthma,” “atopic dermatitis,” “rash,” “hay fever,” “allergic rhinitis,” “allergic conjunctivitis,” “food allergy,” “urticaria,” “SBS,” “multiple chemical sensitivity (MCS),” and “mental illness”], “smoking” history, a history of exposure to cigarette smoke, the existence of a newly built residence or workplace, the number of people living at the place of residence, whether living with children, the disease history of family members living together, “sensitivity of family members living together to air quality and odors,” and the “smoking” history of family members living together. Questions regarding symptoms concerned general symptoms and mucosal irritation symptoms (eye, nasal, and throat symptoms) [28, 29] based on the symptom categories defined by MM040EA, the Japanese version of the MM (Miljumedicin in Swedish) Questionnaire [1]. Specifically, general symptoms included “headache,” “heaviness in the head,” and “tinnitus” on entering the building, and mucosal symptoms included “eye irritation,” “itchy eye,” “dry eye,” “itchy nose,” nasal congestion, rhinorrhea, itchy throat, sore throat, dry mouth, “coughing,” and “sneezing.” Questions regarding residential environment included the number of years lived in the house, the age of the house, the structure of the house, the “material of bedroom window frames,” the “insulating glass pane in the bedroom,” the presence of “condensation,” the presence or absence of “moisture and mold,” odor status, the frequency of “use of deodorant and fragrance” in the house or workplace, the presence or absence of “pets,” the frequency with which windows are opened for “ventilation,” the “frequency of cleaning,” and the location of residence. Questions regarding respondents’ awareness of air quality included the presence or absence of an “interest in indoor air quality and odor” and of “SBS.” In addition, the air-tightness and thermal insulation performance of residential environments were estimated from the type of window sashes and the number of panes of each window [30]. The type of window sash was scored as follows: 1 point for a “wooden” fitting, 2 points for a regular “aluminum sash,” 3 points for an “aluminum double sash,” and 4 points for an “insulation sash.” The type of window was scored as follows: 1 point for “single-pane glass,” 2 points for a “double-pane glass,” and 3 points for a “triple-pane glass.” The thermal insulation performance of windows was defined based on the combined points as follows: 2 points as prior to the S55 standard (energy-saving standard in 1980), 3–4 as the S55 standard, 5 as the H4 standard (The new energy-saving standard), and 6–7 as the H11 standard (the next-generation energy-saving standard) [31].

The protocol of this study was approved by the Research Ethics Committee of the Graduate School of Medicine, School of Medicine, Chiba University (Approval No. 2737).

### Definition of pre-SBS

In this study, we hypothesized that the participants experience SBS-specific symptoms but are not diagnosed either with “SBS” or with “MCS” or that the participants do not seek medical attention due to unawareness of the disease. The lack of awareness due to mild symptoms or exposure to low concentrations of TVOC may lead to exposure for an extended period, ultimately causing severe symptoms and increasing the risk of developing “SBS” [32, 33]. Therefore, those who answered “occasionally,” “often,” or “always” for experiencing at least one of the symptom categories, when they entered their house building, were considered to have a high risk for “SBS” (pre-SBS group).

### Statistical analysis

To identify the factors linked to pre-SBS, a binomial logistic regression analysis using “pre-SBS” as the objective variable was performed. A total of 1493 participants were included in the analysis after excluding 7 individuals who were previously diagnosed with either “SBS” or “MCS.” Multivariate analysis was performed with values of *p* < 0.05 being considered as significant; odds ratios and 95% confidence intervals were also calculated. The presence or absence of multicollinearity between explanatory variables was confirmed using Spearman’s rank correlation analysis; “rearing a child (children)” and “number of people living together” were the only variables that showed a correlation coefficient of ≥ 0.4 at *p* < 0.05. Then, in model 1, the forced entry method was used to analyze the following 18 explanatory variables: sex, age range, years of education, “family income,” “asthma,” “atopic dermatitis,” ‘rash,” “hay fever,” “allergic rhinitis,” “allergic conjunctivitis,” “food allergy,” “urticaria,” “mental illness,” “smoking” history, the existence of a newly built residence or workplace, “interest in air quality and odor,” and the recognition of “SBS” and related diseases. In model 2, the forced entry method was used to analyze 12 items which were used as explanatory variables that related to participants’ residential environments: the disease history of family members living together; the “sensitivity of family members living together to chemical substances or odors;” the location of residence, the years of residence in their current house; the building structure of the house; insulation performance standard; the “condensation status of the house;” the conditions of “moisture and musty odors” in the house; the frequency of “use of deodorant and fragrance;” the presence or absence of “pets;” open-window “ventilation;” and “cleaning frequency.” In model 3, the analysis was performed by the stepwise method (likelihood ratio) using a total of 27 items comprising participant characteristics, awareness, and personal environment as explanatory variables and all other factors as adjustment variables, after determining the influence of factors concerning both a participants’ characteristics and awareness and their personal environment on the development of pre-SBS. The statistical software package SPSS, version 25.0 for Mac (SPSS Inc., Chicago, IL, USA), was used for all analyses.

## Results

### Participants’ characteristics

Participants’ characteristics are shown in Table 1, and the key features are described below.

**Table 1:** Participants’ characteristics

		Number	%			Number	%
Sex				Having a child			
Male		750	50.0	Yes		876	58.4
Female		750	50.0	Family medical history			
Age (years)				SBS		3	0.2
20–29		68	4.5	MCS		1	0.1
30–39		266	17.7	Asthma		95	7.7
40–49		533	35.5	Atopic dermatitis		122	9.8
50–59		496	33.1	Rash		21	1.7
60–69		137	9.1	Hay fever		335	27.0
Education				Allergic rhinitis		186	15.0
< 6 years		6	0.4	Allergic conjunctivitis		34	2.7
6–9 years		69	4.6	Food allergy		40	3.2
10–12 years		454	30.3	Urticaria		74	6.0
≥ 13 years		966	64.4	Mental illnesses		34	2.7
Other		5	0.3	Sensitivity of family to air quality and odor			
Family income (yen)				Yes		373	24.9
< 3 million		329	21.9	Family smoking			
3–< 6 million		534	35.6	Yes		654	43.6
6–< 9 million		346	23.1	Duration of residence			
9–< 15 million		231	15.4	< 5 years		268	17.9
≥ 15 million		60	4.0	5–< 10 years		283	18.9
Medical history				10–< 20 years		461	30.7
SBS		1	0.1	≥ 20 years		488	32.5
MCS		6	0.4	Age of house building			
Asthma		134	8.9		<5 years	103	6.9
Atopic dermatitis		130	8.7		5–< 10 years	158	10.5
Rash		56	3.7		10–< 20 years	423	28.2
Hay fever		386	25.7		≥ 20 years	816	54.4
Allergic rhinitis		244	16.3	House construction			
Allergic conjunctivitis		65	4.3	Wooden		729	48.6
Food allergy		47	3.1	Material window flame (bedroom)			
Urticaria		154	10.3	Old wooden sash		28	1.9
Mental illnesses		76	5.1	Aluminum sash		853	56.9
Smoking				Double aluminum sash		260	17.3
Never		794	52.9	Insulation sash		144	9.6
Quit		340	22.7	Others		215	14.3
Current		366	24.4	Insulating glass pane type (bedroom)			
Exposure to tobacco				Single		884	58.9
Never		150	10.0	Double		427	28.5
Rare		236	15.7	Triple		11	0.7
Sometimes		567	37.8	Others		178	11.9
Often		547	36.5	Presence of condensation			
Living experience in a renovated house/new house				Yes		1020	68.0
Yes		1004	66.9	Moisture and musty odor			
Headache, heaviness in head, tinnitus				Yes		786	52.4
Never		1185	79.0	Using deodorant and fragrance			
Rare		193	12.9	No		392	26.1
Sometimes		78	5.2	Rare		344	22.9
Often		24	1.6	Sometimes		439	29.3
Always		20	1.3	Often		196	13.1
Itching, burning, irritation, drying of the eyes				Every day		129	8.6
Never		1189	79.3	Pet			
Rare		201	13.4	Yes		429	28.6
Sometimes		82	5.5	Ventilation			
Often		13	0.9	Less than once/day		391	26.1
Always		15	1.0	More than once/day		1109	73.9
Irritated, stuffy, or runny nose and irritated or dry throat				Frequency of cleaning			
Never		1023	68.2	Less than once/week		337	22.5
Rare		279	18.6	More than once/week		1163	77.5
Sometimes		145	9.7	Residential area			
Often		38	2.5	Hokkaido		59	3.9
Always		15	1.0	Tohoku		74	4.9
Cough and sneezes				Kanto		595	39.7
Never		994	66.3	Chubu		236	15.7
Rare		311	20.7	Kansai		329	21.9
Sometimes		152	10.1	Chugoku		80	5.3
Often		30	2.0	Shikoku		42	2.8
Always		13	0.9	Kyushu, Okinawa		85	5.7
Number of family members				Interest in air quality and odor			
1		261	17.4	Yes		958	63.9
2		422	28.1	Knowledge regarding SBS			
3		388	25.9	No		128	8.5
4		302	20.1	Have heard		778	51.9
≥ 5		127	8.5	Aware		464	30.9
				Deep knowledge		130	8.7

The age range of participants was as follows: 20–29 years, 4.5%; 30–39 years, 17.7%; 40–49 years, 35.5%; 50–59 years, 33.1%; and 60–69 years, 9.1%. As “disease history,” 0.1% of participants had “SBS,” 0.4% had “MCS,” and 25.7% had “hay fever” (the last of which was the most frequent condition to appear in respondents’ medical histories). For “smoking” history, 22.7% of respondents reported having previously smoked but were not currently “smoking,” and 24.4% reported that they were currently “smoking.” For symptoms such as “headache,” “heaviness in the head,” and “tinnitus” that appeared upon entering their building, 12.9%, 5.2%, 1.6%, and 1.3% respondents answered “rarely,” “occasionally,” “often,” and “always,” respectively. With regard to mucosal symptoms, 13.4–20.7% answered “rarely,” 5.5–10.1% answered “occasionally,” 0.9–2.5% answered “often,” and 0.9–1.0% answered “always.” In response to the question regarding personal environment, 24.9% of respondents had “a family member living together with sensitivity to chemicals and odor.” Moreover, 48.6% lived in a “wooden” house based on responses to the question regarding the “building structure of the house.” Regarding the “condensation” status of the house, 68% answered that “condensation” existed, and 52.4% reported the presence of “moisture and musty odor.” Regarding the frequency of “use of deodorant and fragrance,” 26.1% of respondents reported not using them at all, 22.9% reported rare use, 29.3% reported occasional use, 13.1% reported frequent use, and 8.6% reported daily use. Regarding the frequency of opening windows for “ventilation”, 73.9% answered that they did this “more than once/day,” and as for “frequency of cleaning,” 77.5% answered that they cleaned “more than once/week.” As for awareness, 63.9% of respondents had an “interest in air quality and odor,” while with regard to “recognition of SBS,” 51.9% had heard about it, 30.9% knew about it, and 8.7% knew it well.

### Pre-SBS

Table 2 shows the prevalence and characteristics of the pre-SBS group and the features are described as follows. The answers for “occasionally,” “often,” and “always” for each symptom accounted to 120 (8.0%) for “headache,” 108 (7.2%) for eye symptoms, 195 (13.1%) for nasal symptoms, and 192 (12.9%) for airway symptoms. One thousand two hundred eighteen people among all the participants answered that they had no or rare symptoms and they were judged as those at low risk of “SBS.” A total of 282 participants (18.8% of the total participants; male 45.4%, female 54.6%) in this study had at least one symptom and they were considered as a high-risk group and defined as pre-SBS symptoms.

**Table 2:** Prevalence and characteristics of Pre-SBS group

	Group at low risk of SBS, *n* (%)		Group at high risk of SBS, *n* (%) = pre-SBS, 282 (18.8)			
	Never	Rare	Sometimes	Often	Always	Total
Headache, heaviness in head, tinnitus	1181 (79.1)	192 (12.9)	78 (5.2)	22 (1.5)	20 (1.3)	120 (8.0)
Itching, burning, irritation, drying of the eyes	1187 (79.5)	198 (13.3)	81 (5.4)	13 (0.8)	14 (0.9)	108 (7.2)
Irritated, stuffy, or runny nose	1020 (68.3)	278 (18.6)	144 (9.7)	37 (2.5)	14 (0.9)	195 (13.1)
Cough and sneezes	991 (66.4)	310 (20.7)	152 (10.2)	28 (1.9)	12 (0.8)	192 (12.9)

### Characteristics of the pre-SBS group

The results of the binomial logistic regression analysis for participants in the pre-SBS group using objective variables are shown in Table 3, and the characteristic features are described below.

**Table 3:** Results of univariate and multivariate regression analyses for the association of objective variables with pre-SBS

	Charactristics			Environment			Charactristics + Environment		
	Adjusted OR	95% CI		Adjusted OR	95% CI		Adjusted OR	95% CI	
Sex									
Male	0.96	0.71	1.30						
Age									
20-29	Ref.						Ref.		
30-39	0.62	0.33	1.15				0.50	0.22	1.11
40-49	0.50*	0.27	0.90				0.46*	0.21	0.99
50-59	0.26***	0.14	0.50				0.26**	0.12	0.57
60-69	0.20***	0.09	0.45				0.25**	0.10	0.64
Medical history									
Asthma	1.18	0.75	1.86						
Atopic dermatitis	0.84	0.52	1.35						
Rash	1.08	0.55	2.14						
Hay fever	1.35	0.99	1.83				1.58**	1.14	2.19
Allergic rhinitis	2.52***	1.76	3.62				2.63***	1.85	3.74
Allergic conjunctivitis	0.73	0.39	1.39						
Food allergy	1.61	0.81	3.21						
Urticaria	1.07	0.69	1.67						
Mental illnesses	2.25**	1.31	3.86				2.07*	1.16	3.68
Smoking									
Never	Ref.								
Quit	1.15	0.81	1.63						
Current	1.02	0.71	1.47						
Interest in air quality and odor	1.51*	1.10	2.08						
Sensitivity of family members to VOCs and odor				2.32***	1.69	3.18	2.11***	1.53	2.92
Duration of residence									
< 5 years				Ref.					
5 -< 10 years				0.79	0.49	1.27			
10 -< 20 years				0.59*	0.38	0.92			
≥ 20 years				0.53**	0.34	0.84			
Condensation in winter				1.61**	1.10	2.36	1.59*	1.09	2.34
House construction (excluding wooden houses)				1.20	0.88	1.63			
Therma insulation performance									
Prior to the S55 standard				Ref.					
Standard S55				1.15	0.61	2.19			
Standard H4				1.09	0.61	1.94			
Standard H11				1.85	0.90	3.78			
Moisture and musty odor				1.97***	1.41	2.74	1.73**	1.23	2.43
Deodorant and fragrance use									
No				Ref.			Ref.		
Rare				0.81	0.51	1.29	0.75	0.46	1.22
Sometimes				1.26	0.83	1.91	1.17	0.76	1.79
Often				1.65*	1.07	2.54	1.46	0.93	2.28
Ventilation less than once/day				1.11	0.81	1.52			
Cleaning frequency less than once/week				1.54*	1.06	2.23	1.60*	1.11	2.31

significant at *p<.05, **p<.01,***p<.001

In model 1, in which individual characteristics and awareness were the adjustment variables, a significant association was observed for four variables at *p* < 0.05. Regarding age groups, based on the youngest group who was age of 20–29 years as the reference, the proportion of participants with pre-SBS in each age group decreased with increasing age: OR for the participants aged 40–49, 50–59, and 60–69 years were 0.50, 0.26, and 0.20, respectively. In addition, with regard to disease history, the probability of having pre-SBS was higher in those with a history of “allergic rhinitis” (OR = 2.52) or “mental illness” (OR = 2.25). Regarding awareness, the probability of pre-SBS was higher in those with the “interest in air quality and odor” (OR = 1.51). In model 2, in which residential environment was an adjustment variable, a significant association was observed for six variables at *p* < 0.05. Particularly, having “a family member with sensitivity to chemical substances and odor” was associated with a high probability of pre-SBS (OR = 2.32). Regarding “duration of residence in the house” using < 5 years as a reference, a higher “duration of residence” was inversely associated with a higher probability of pre-SBS, indicated by 10–< 20 years (OR = 0.59) and ≥ 20 years (OR = 0.53). The results also showed that a higher probability of pre-SBS was associated with “condensation status of the house” (OR = 1.61) and “moisture and musty odor” (OR = 1.97). In addition, regarding the frequency of “use of deodorant and fragrance,” when the response of “not at all” was set as the reference, the probability of pre-SBS associated with “use every day” increased (OR = 1.65). Conversely, regarding “frequency of cleaning,” when “cleans everyday” was set as the reference, the OR for “less than once/week” was 1.54, indicating a high probability of pre-SBS. The other eight items had a significant relationship with pre-SBS in the final model (model 3), with individual characteristics, awareness, and personal environment as adjustment variables. In particular, the probability of pre-SBS declined with increasing age. However, for medical history, in addition to having a history of “allergic rhinitis” and “mental disorder,” “hay fever” was associated with a high probability of having pre-SBS (OR = 1.58). Conversely, “interest in air quality and odor,” “duration of residence in the current house,” or frequency of “use of deodorant and fragrance” did not show any significant associations following adjustment.

## Discussion

This study revealed that 18.8% of all respondents were categorized as pre-SBS based on their experience of “headache” or any symptoms in their eyes, nose, or throat upon entering their current building. Many previous reports have indicated that females are more likely to experience “SBS” or hypersensitivity to chemical substances [4, 34, 35]. There are various reasons for this, including females’ generally higher awareness of health [34], a strong feeling of repulsion in response to certain odors [7], and a high exposure to TVOC of females compared with males [8]. The prevalence of “SBS” is higher among females compared with males, regardless of individual, occupational, and building-related factors [36]. However, the current study revealed that the percentage of females and males was 54.4% and 45.6%, respectively, in the pre-SBS group, which includes individuals who are not diagnosed with “SBS” or “MCS” but who experienced symptoms that were not severe enough to be recognized as symptoms of these conditions. This indicates that males have SBS-specific experiences similar to females. With regard to the age of participants in the pre-SBS group, the highest occurrence of symptoms was observed in younger participants (aged 20–29 years) and 0.5 times in those aged 40–49 (*p* < 0.05), 0.3 times in those aged 50–59 (*p* < 0.001), and 0.2 times in those aged 60–69 (*p* < 0.001), indicating a significant decrease with increasing age. Previous studies regarding “MCS” and allergies have reported various characteristics regarding age. One study reported a higher occurrence of these conditions in individuals aged < 30 years [37], and another study reported that these conditions were observed in individuals of all ages [4]. These results which are different from those of the previous study may indicate that certain men and women, including young and old people, are equally sensitive to chemical exposure. Just the number of people who are interested and aware of the symptoms may be different, and those who are interested may be taking action to avoid the risk factors in the environment. This is a limitation of this cross-sectional study, and further investigation is needed. However, “SBS” and “MCS” can develop as diseases following exposure chemical substances, even to low concentrations if the exposure is continuous [32, 33]. Alternatively, the young participants who comprised a high percentage of the pre-SBS group in the current study have a longer life expectancy, so they may suffer from worsening symptoms or the onset of diseases due to exposure over a long period if they remain unaware of their symptoms. This may increase the number of patients with these conditions in the future. In addition, further attention should be paid to the relationship between other diseases and chemicals in the air, not just “SBS” and “MCS” [38–40].

Analysis using individual characteristics as adjustment variables (model 1) revealed the significant association between the pre-SBS group and a history of “allergic rhinitis,” a history of “mental illness,” and the presence or absence of an “interest in air quality and odor.” Many studies have reported a relationship between a history of these diseases (allergies and “mental illness”) and “SBS” and “MCS.” For example, one report has suggested that the estimated prevalence of “MCS” is higher in patients with allergic diseases than in patients who did not have allergic diseases [37]. There is another report that indicated similar results on the association between psychological factors and “SBS” and “MCS” [16] which were observed in the pre-SBS group in this study. Regarding the personal environment, including the residential environment, showed an association with pre-SBS; a significant relationship was seen between the answer of “always” to questions relating to “condensation” status in the house, “moisture and musty odors,” frequency of “use of deodorant and fragrance,” and insulation efficiency of the house. According to previous studies, the prevalence of “SBS” was higher among individuals living in unsanitary buildings compared with those living in clean buildings [18, 32, 41, 42]; the impact of mold [41–45] and “ventilation” on health has also been reported [46, 47]. The current study indicated that pre-SBS showed patterns similar to those in the abovementioned studies. On the contrary, there was no direct relationship between highly airtight, new buildings and “wooden” houses and pre-SBS. However, considering the result that a residential environment with “condensation” and “moisture/mold” and lifestyle habits such as “frequency of cleaning” of “less than once/week” and heavy fragrance use could lead to an increased risk of pre-SBS, it is clear that individuals should be aware that they should maintain good air quality in their daily life and that living spaces with structures capable of maintaining a continuous, high-quality air environment are both important. To prevent health hazards arising from indoor air and to enjoy a safe and healthy life, it is important to enlighten people and encourage them to regularly clean and ventilate their living spaces, in addition to planning healthy residential environments with mechanical ventilation systems as well as efficient natural ventilation to prevent air retention in buildings. There are some limitations in this study. (1) Causal relationships could not be provided in this study because of the cross-sectional design. (2) This study may be subject to recall bias because the health outcomes, participants’ characteristics, and environmental factors used in the assessment in this study were collected on a questionnaire basis and were subjective to the respondents. (3) There may be selection bias, and the participants may not present the population because this survey was registered by an internet survey site.

## Conclusions

We categorized females and males (age, 20–69 years) who had experienced specific symptoms of “SBS” “occasionally,” “often,” or “always” even without a diagnosis of “SBS” in the pre-SBS group. We then investigated and analyzed associated factors, such as individual characteristics, residential environment, and participants’ awareness of relevant facts. Results revealed that the risk of pre-SBS was higher in younger participants, regardless of sex, indicating that it is important to create a good indoor environment. Although recently built, highly airtight and highly insulated “wooden” housing did not show a tendency toward directly increasing the risk of pre-SBS. Maintaining a good indoor environment reduces the risk of pre-SBS; therefore, in addition to educating people, air conditioning and ventilation plans should always ensure a healthy indoor environment.

## Data Availability

The datasets generated during and/or analyzed during the current study are not publicly available due to joint research and development with the company but are available from the corresponding author on reasonable request.

## Abbreviations

SBS: Sick building syndrome
VOCs: Volatile organic compounds
MCS: Multiple chemical sensitivity
